# The role of adipokines in skeletal muscle inflammation and insulin sensitivity

**DOI:** 10.1186/s12950-018-0185-8

**Published:** 2018-05-09

**Authors:** Thomas Nicholson, Chris Church, David J. Baker, Simon W. Jones

**Affiliations:** 1 MRC-ARUK Centre for Musculoskeletal Ageing Research, Medical School, Queen Elizabeth Hospital, University of Birmingham, Birmingham, B15 2WB UK; 2 MedImmune, Cardiovascular and Metabolic Disease (CVMD), Milstein Building, Granta Park, Cambridge, CB21 6GH UK; 3 Institute of Inflammation and Ageing, MRC-Arthritis Research UK Centre for Musculoskeletal Ageing Research, Queen Elizabeth Hospital, Mindelsohn Way, Edgbaston, Birmingham, B15 2TT UK

**Keywords:** Obesity, Adipokines, Inflammation, Skeletal muscle, Myotubes, Insulin signalling, Type II diabetes

## Abstract

**Background:**

There is currently an unmet clinical need to develop better pharmacological treatments to improve glucose handling in Type II Diabetes patients with obesity. To this end, determining the effect of obesity-associated adipokines on skeletal muscle insulin sensitivity has emerged as an important area of drug discovery research. This review draws together the data on the functional role of adipokines on skeletal muscle insulin signalling, highlights several understudied novel adipokines and provides a perspective on the direction of future research.

**Main body:**

The adipokines leptin, resistin, visfatin and adiponectin have all been shown to affect skeletal muscle insulin sensitivity by impacting on the activity of components within insulin signalling pathways, affecting GLUT4 translocation and modulating insulin-mediated skeletal muscle glucose uptake. Furthermore, proteomic analysis of the adipose tissue secretome has recently identified several novel adipokines including vaspin, chemerin and pref-1 that are associated with obesity and insulin resistance in humans and functionally impact on insulin signalling pathways. However, predominantly, these functional findings are the result of studies in rodents, with in vitro studies utilising either rat L6 or murine C2C12 myoblasts and/or myotubes. Despite the methodology to isolate and culture human myoblasts and to differentiate them into myotubes being established, the use of human muscle in vitro models for the functional validation of adipokines on skeletal muscle insulin sensitivity is limited.

**Conclusion:**

Understanding the mechanism of action and function of adipokines in mediating insulin sensitivity in skeletal muscle may lead to the development of novel therapeutics for patients with type 2 diabetes. However, to date, studies conducted in human skeletal muscle cells and tissues are limited. Such human in vitro studies should be prioritised in order to reduce the risk of candidate drugs failing in the clinic due to the assumption that rodent skeletal muscle target validation studies will to translate to human.

## Background

Type II diabetes (T2D) is a chronic metabolic disorder that carries a significant and increasing economic burden [1]. Unfortunately, there is no cure for T2D and treatments are limited. Furthermore, the inability of patients to maintain plasma glucose concentrations is associated with a number of chronic pathologies, including microvascular disease and macrovascular diseases such as stroke and coronary artery disease [2].

One of the major treatment strategies for T2D patients is to increase insulin sensitivity, either through lifestyle modifications such as weight loss, or via the administration of insulin-sensitising drug therapies including Biguanides such as Metformin [3, 4] and Thiazolidinediones [5]. Alternatively, some patients are prescribed Sulphoylureas, which stimulate insulin secretion [6, 7]. However, these medications are associated with significant side-effects when taken chronically and can become ineffective as disease progresses [8–13]. Therefore, there is great unmet clinical need to develop more effective and more targeting therapeutics for T2D patients.

In attempting to identify new therapies, skeletal muscle has emerged as an important area of drug discovery research. Muscle metabolic function is considered central to maintaining insulin sensitivity [14, 15], being responsible for up to 80% of insulin-mediated glucose uptake in healthy individuals [16]. Indeed, T2D patients display impaired skeletal muscle glucose uptake in response to insulin. Given the association of obesity and T2D and the paradigm of adipose tissue as an endocrine organ [17–19], recent studies have examined the cross-talk between skeletal muscle and adipose tissue in the context of insulin resistance. In obese individuals, adipose tissue is known to become more “inflammatory”, with an increase in the infiltration of immune cells including T-cell subsets [20] and inflammatory M1 macrophages [21], which drive the production of pro-inflammatory adipokines associated with insulin resistance [22, 23].

Importantly, secretome analysis of human adipocyte culture medium has identified over 200 adipokines [24]. Therefore, understanding the functional and mechanistic role of these adipokines on skeletal muscle insulin signalling could identify novel targets for therapeutic intervention. Here we summarise the key studies conducted to date on the functional role of adipokines on skeletal muscle insulin signalling, highlight several understudied novel adipokines and provide a perspective on the direction of future research.

### Established well known Adipokines

The adipokines leptin, adiponectin, resistin and visfatin are known mediators of inflammation and have all been implicated in metabolic diseases, including T2D. Below, we highlight studies conducted on these adipokines which relate to their functional role in skeletal muscle insulin signalling, and summarise these findings in Table 1 in relation to animal and human data.

**Table 1:** Evidence for the role of known adipokines in mediating skeletal muscle insulin sensitivity

Adipokine	Association with obesity and/or T2D in humans	Adipokine effect on insulin signalling in animal models		Adipokine effect on insulin signalling in human skeletal muscle
		In Vivo	In Vitro	
Leptin	Increased [25, 26, 127].	Overexpression of leptin in a skinny mouse model increased insulin sensitivity [29]. Administration of leptin (12–15 days) reversed insulin resistance in obese wistar rats [128]. Leptin reversed high fat diet induced skeletal muscle insulin resistance in rats, indirectly via reducing intramuscular triglycerides not though direct modulation of insulin signalling [129].	Recombinant leptin reduces IRS-1 phosphorylation and glucose uptake in L6 myotubes [27].	Increased phosphorylation of AKT in commercially available primary human myotubes [30].
			Recombinant leptin increased glucose uptake in C2C12 myotubes [28]. Acute (10mins-1 h) stimulation of L6 Myotubes directly increased glucose uptake via a PI3K-dependent pathway. Leptin pre-treatment (10 min) of L6 myotubes inhibits insulin stimulated glucose uptake [130]. 24 h Pre-treatment of L6 myotubes had no effect on glucose uptake but did inhibit adiponectin stimulated glucose uptake [131].	
Adiponectin	Decreased [31, 32].	Adiponectin knockout mice demonstrate an obese and insulin resistant phenotype [37, 39].	Promotes glucose uptake in both C2C12 and L6 Myotubes [35, 36].	Induces fat oxidation through activation of AMPK in myotubes from lean subjects. Mechanism impaired in myotubes from T2D patients [41].
		Systemic administration and overexpression of adiponectin drives increased insulin sensitivity in insulin resistant mice [38, 40].	Recombinant adiponectin increased glucose uptake via AMPK mediated reorganisation of the actin cytoskeleton and GLUT4 translocation via an independent mechanism [130].	
Resistin	Increased [45, 46].	Administration of resistin (6 days) to wild type mice induces a state of insulin resistance [132].	Recombinant resistin Impaired insulin signalling and glucose uptake in both C2C12 and L6 myotubes [48, 49].	Unknown
		Targeted reduction of resistin in insulin resistant mice via antisense oligodeoxynucleotide restored hepatic but not skeletal muscle insulin sensitivity [133].		
Visfatin	Increased [134–136].	Visfatin overexpression in rats increased whole body insulin sensitivity and adipose tissue and liver IRS-1 phosphorylation in response to insulin [56].	Stimulated glucose uptake and increased GLUT4 membrane translocation and mRNA and protein expression in C2C12 myotubes via AMPK p38 MAPK signalling [57].	Unknown
			Increased glucose uptake in rat EDL muscle [137].	

### Leptin

The role of leptin as an inflammatory adipokine in metabolic disorders is well studied. Systemic levels of leptin positively correlate with both BMI and waist circumference, and are associated with the development of insulin resistance [25, 26]. Several studies have reported that leptin impacts on skeletal muscle insulin signalling. Stimulation of the rat L6 skeletal muscle cell line with recombinant leptin reduced phosphorylation of the insulin receptor substrate-1 (IRS-1) and impaired glucose uptake, suggesting that leptin promotes insulin resistance [27]. However, in contrast, leptin stimulation of murine C2C12 myotubes was found to increase glucose uptake, whilst overexpression of leptin in a ‘skinny’ mouse model increased insulin sensitivity [28, 29]. These contrasting data highlight the need to conduct functional studies on leptin in human myotubes. To this end, Yau et al. reported that leptin increased AKT phosphorylation in commercially available human myotubes [30]. However, to date the functional role of leptin on human skeletal muscle insulin signalling is still understudied.

### Adiponectin

Adiponectin is considered to be a beneficial adipokine in relation to metabolism; plasma concentrations inversely correlate with weight, central obesity, risk of T2D and insulin resistance in humans [31, 32]. Furthermore, maintenance of a low calorie intake increases both adipocyte expression of adiponectin and circulatory concentrations [33].

Three different molecular weight isoforms of adiponectin are found in the circulation, of which the high molecular-weight isoform is beloved to be the most functional in terms of glucose homeostasis. Functional studies suggest that adiponectin promotes insulin sensitivity in skeletal muscle. In C2C12 myocytes, adiponectin increases fatty acid oxidation via sequential activation of AMPK, p38 MAPK and PPARα [34, 35] and promotes glucose uptake [35]. Similarity, in L6 myotubes adiponectin induces glucose transporter 4 (GLUT4) translocation and glucose uptake [36]. In vivo, adiponectin knockout mice demonstrate an obese, insulin resistant phenotype, whereas systemic administration of adiponectin, or its delivery as a transgene direct to skeletal muscle, improves insulin sensitivity [37–40]. Adiponectin has also been shown to induce fat oxidation via AMPK activation in human myotubes, and further, this mechanism was found to be impaired in myotubes from obese T2D patients [41]. Critically, this suggests that the function of adiponectin as a promoter of insulin sensitivity translates to humans. Furthermore, it suggests that impairment of adiponectin function in skeletal muscle of obese T2D patients may contribute in the development of insulin resistance.

### Resistin

First identified in murine adipocytes as a secreted protein capable of inducing insulin resistance [42], resistin is a pro-inflammatory adipokine that induces the secretion of TNFα and IL-6 from various cell types including PBMCs and pancreatic acinar cells [43, 44].

The correlation between plasma resistin with both obesity and insulin resistance in humans support a role for resistin in the development of insulin resistance [45, 46]. In vitro, studies have demonstrated a reduction in AKT phosphorylation and glucose uptake in C2C12 and L6 myotubes stimulated with recombinant resistin [47–49]. However, at present few studies have investigated the functional role of resistin in the development of insulin resistance in human skeletal muscle cells.

### Visfatin

Visfatin (also called NAMPT) is termed an ‘adipokine-enzyme’ due to the NAD biosynthesis function of its intracellular form (iVisfatin/iNAMPT), also exists extracellularly (eVisfatin/eNAMPT) [50, 51]. eVisfatin is primarily produced and secreted from visceral adipose tissue, where it is more highly expressed in obese individuals [52]. Similarly, higher systemic levels of evisfatin are associated with obesity, ageing and the development of T2D [53–55].

Regarding the role of visfatin in mediating insulin sensitivity, overexpression of visfatin in male wistar rats increased whole body insulin sensitivity [56], and in adipose tissue and liver, promoted insulin-mediated IRS-1 phosphorylation [56]. Data on the function of visfatin in skeletal muscle insulin sensitivity is limited to studies in rodents. Visfatin increases glucose transport in rat skeletal muscle fibres [57]. Furthermore, in C2C12 myotubes, visfatin activates AMPK/p38 MAPK, induces GLUT4 expression and translocation, and promotes glucose uptake [57]. Based on these data, similar insulin sensitizing effects may occur in human skeletal muscle.

### Novel Adipokines

In addition to the well-known adipokines, proteomic studies of adipose tissue have identified several less characterised adipokines that may also play important roles in mediating skeletal muscle insulin sensitivity. At present the functional effects of the majority of these novel adipokines on human skeletal muscle insulin sensitivity s poorly understood. Some of the more prominent novel adipokines are discussed below and also summarised in Table 2.

**Table 2:** Evidence for the role of novel adipokines in mediating insulin sensitivity

Adipokine	Association with obesity and/or T2D in humans	Adipokine effect on insulin signalling in animal models		Adipokine effect on insulin signalling in human skeletal muscle
		In Vivo	In Vitro	
FGF-21	Increased [86].	Increased insulin sensitivity and glucose uptake in mice, via FGF-21 mediated increases in adiponectin production and secretion from adipocytes [76].	6 h incubation of mouse EDL muscle with FGF-21 resulted in a 54% increase in insulin stimulated glucose uptake [86].	Directly increased glucose uptake in primary human myotubes [86]. Prevents palmitate-induced insulin resistance in primary human myotubes by inhibiting stress kinases and NF-κB [87].
		Continuous cerebral administration for 2 weeks increased whole body insulin sensitivity in rats with dietary induced obesity [77].		
		Daily administration for 6 weeks improved glucose handling in diabetic rhesus monkeys [78].		
Chemerin	Increased [94, 138].	Overexpression increased insulin resistance in LDL receptor deficient mice by reducing AKT phosphorylation in response to insulin in skeletal muscle, but not liver or pancreas [96].	24 h pre-treatment reduces insulin stimulated glucose uptake in C2C12 myotubes in a dose dependent manor [99].	24 h chemerin Increased insulin resistance and reduced insulin stimulated glucose uptake in primary human myotubes, mediated by increased ERK signalling [95].
		knockout mice display increased skeletal muscle insulin resistance while transgenic mice exhibit increased skeletal muscle insulin resistance [98].		
		Acute chemerin treatment exacerbated glucose intolerance and lowered serum insulin levels in obese and diabetic mice. No effect observed in normoglycemic mice [97].		
CTRP3	Decreased [115, 116, 139].	Administration of recombinant CTRP3 directly lowers glucose levels in normal and insulin-resistant ob/ob mice [140].	Administration of recombinant CTRP3 to L6 myotubes had no effect on glucose uptake [140].	Unknown
		Overexpression of CTRP3 improved insulin sensitivity in HFD fed mice [141].	Increased glucose uptake and GLUT 4 mRNA expression in insulin resistant adipocytes [142].	
RBP4	Increased [143, 144].	Overexpression or direct administration of RBP4 increased insulin resistance in mice. RBP4 knockout improves insulin sensitivity in mice [144].	unknown	Unknown
		Reducing circulating RBP4 in obese mice models improved glucose tolerance and increased insulin stimulated glucose uptake in skeletal muscle up to 60% [145].		
Vaspin	Increased [65, 67, 68].	Vaspin treatment increased insulin sensitivity and glucose tolerance in obese and diabetic mice [59, 60].	Unknown	Unknown
		transgenic mice overexpressing vaspin displayed improved glucose tolerance and were protected from obesity when challenged with a high fat diet [62].		
Pref-1	Increased [101].	Overexpression in mice drives insulin resistance via decreased adipose tissue and skeletal muscle glucose uptake and impaired skeletal muscle insulin signalling [105].	Unknown	4 Day exposure to primary human myotubes from lean, obese and T2D subjects had no effect on glucose uptake [106].
Follistatin-like 1	Increased [108].	Unknown	Blunts insulin signalling-adipocytes [108].	unknown
Omentin-1	Decreased [146, 147].	Unknown	omentin-1 induced AKT phosphorylation and enhanced insulin-stimulated glucose uptake in human adipocytes [123].	Unknown Unknown
Lipocallin-14	Unknown	Over expression in diet induced obese mice reduced glucose and insulin levels while improving glucose tolerance [124].	Unknown	

### Vaspin

First reported as a 47KDa protein in the visceral adipose tissue of the genetically obese OLETF rats [58], administration of vaspin to obese mice increased insulin sensitivity and glucose tolerance [59]. Additionally, subcutaneous adipose tissue expression of leptin, resistin and TNFα was suppressed, whilst GLUT4 and adiponectin expression was increased following vaspin administration [59]. Similar increases in insulin sensitivity have since been reported in db/db and C57BL6 mice following recombinant vaspin delivery [60]. Central administration of vaspin to obese mice resulted in a sustained suppression of appetite that resulted in reduced bodyweight and plasma glucose concentrations [61]. Furthermore, transgenic mice overexpressing vaspin displayed improved glucose tolerance, reduced systemic IL-6 concentrations and were protected from obesity when fed a high fat diet [62].

In humans, vaspin expression has been reported in several tissues including subcutaneous adipose tissue, skin, stomach and skeletal muscle [61, 63, 64]. Serum concentrations of vaspin in non-diabetic and diabetic patients positively correlate with BMI, bodyweight and impaired glucose tolerance [65–68]. Given the functional effects of vaspin demonstrated in the rodent models, its increased expression with BMI in humans may reflect a compensatory mechanism.

The effect of vaspin on insulin signalling and metabolism in human skeletal muscle is currently undetermined. Similarly, the mechanism of action and receptor for vaspin has also not been elucidated. Recently, it was reported that in HepG2 cells vaspin binds glucose-regulated protein (GRP78), a 7KDa voltage-dependent anion channel. Further, stimulation of H-4-II-E-C3 cells with recombinant vaspin activated AKT and AMPK signalling pathways, which was prevented by GRP78 inhibition [62]. Vaspin may therefore mediate its effects on insulin signalling via binding to GRP78. However, at present the expression of GRP78 has not been profiled in human adipose or skeletal muscle tissue, nor the functional studies conducted in human skeletal cells to validate GRP78 as the vaspin receptor.

### Fibroblast growth factor 21

FGF-21 is established as a key mediator of fat oxidation and in energy homeostasis [69–71]. Numerous studies report that serum concentrations of FGF-21 are elevated in obese individuals and positively correlate with insulin resistance, BMI, % fat mass and circulatory concentrations of leptin and LDL [72–75]. Although predominantly produced by the liver, FGF-21 is also expressed in adipose tissue, where it is more highly expressed in both obese and diabetic mouse models.

In vivo, administration of FGF-21 to mice fed a high fat diet decreased intramuscular triglyceride content, increased insulin sensitivity and glucose uptake, and elevated secretion of adiponectin from adipocytes [76]. Continuous cerebral administration of FGF-21 for 2 weeks increased whole body insulin sensitivity in rats with dietary-induced obesity [77], whilst daily intravenous or subcutaneous delivery of FGF-21 for 6 weeks improved glucose handling in diabetic rhesus monkeys [78]. Following such positive effects on insulin sensitivity and glucose tolerance, two FGF-21 mimetics (LY2405319 and PF-05231023) have progressed to phase 1 clinical trials (NCT01869959, NCT01923389) [79–83], and antibodies targeting FGFR1c/b-Klotho have been developed [84, 85].

With regards to a direct functional role of FGF-21 in skeletal muscle, incubation of isolated mouse EDL muscle with FGF-21 increased insulin-stimulated glucose uptake, and in human myotubes FGF-21 increased both basal and insulin-stimulated glucose uptake [86]. Furthermore, FGF-21 has also shown to prevent palmitate-induced insulin resistance in primary human myotubes by inhibiting stress kinases and NF-κB [87].

### Chemerin

Chemerin, was initially described as a novel chemoattractant for macrophages and dendritic cells via activation of several GPCRs including CMKLR1/ChemR23, GPR1, and CCRL2 [88, 89]. More recent data suggests chemerin plays an important role in the differentiation of human adipocytes [90, 91], and in the development of insulin resistance. Circulatory concentrations of chemerin are associated with obesity, diabetes and metabolic syndrome [92–94] . Furthermore, adipose tissue from obese subjects exhibits greater secretion of chemerin [95].

At present, in vivo studies have drawn differing conclusions regarding the role of chemerin in the development of insulin resistance. Becker et al. reported that overexpression of chemerin increased insulin resistance in LDL-receptor deficient mice fed a high fat diet, as evidenced by reduced insulin-mediated AKT phosphorylation [96]. Importantly this effect was only observed in skeletal muscle, and not liver or pancreas [96]. Additionally, glucose handling and serum insulin concentrations were reduced by chemerin administration to both obese and diabetic mice [97]. However, no such effect was observed following chemerin administration to control mice. In contrast, Takahashi et al. showed that chemerin knockout mice display increased skeletal muscle insulin resistance, due to a disruption of hepatic glucose production and reduced insulin secretion from pancreatic Beta cells [98]. Additionally, transgenic mice overexpressing chemerin were reported to have increased skeletal muscle insulin sensitivity [98].

In vitro studies provide support for chemerin as a driver of insulin resistance. Pre-treatment of C2C12 myotubes with chemerin reduced insulin-stimulated glucose uptake, while increasing the secretion of pro-inflammatory cytokines including IL-6 and TNF-α [99]. Additionally, treatment of primary human myotubes with recombinant chemerin reduced insulin-stimulated glucose uptake [95]. Further cross-talk studies with primary human myocytes and myotubes, particularly from obese and diabetic cohorts may help to clarify the function of chemerin in human metabolic disease states.

### Pref-1

Preadipocyte factor 1 (Pref-1) is a transmembrane protein processed to generate a circulating form, which is also known as Foetal Antigen 1 (FA1) [100]. Studies have described an association of increased Pref-1/FA1 serum concentrations with obesity and T2D [101, 102]. Pref-1 is also reported to negatively regulate adipogenesis, with Pref-1 deficient mice displaying significant obesity and stunted growth [103, 104]. Overexpression of Pref-1 in mice promotes a lipodystrophic phenotype and insulin resistance via decreased skeletal muscle glucose uptake and impaired skeletal muscle insulin signalling [105].

In humans, Pref-1 stimulation of myotubes from lean, obese, and T2D patients with did not affect insulin sensitivity. However Pref-1 did induce the production of the pro-inflammatory IL-6 and CCL2 [106], and thus chronic exposure of muscle to pathological levels of Pref-1 may impair insulin sensitivity indirectly. Clearly, further studies utilising human myotubes are warranted to fully determine the functional role of Pref-1 in skeletal muscle insulin sensitivity.

### Follistatin-like 1

Follistatin-like 1 (FSTL1) is a glycoprotein with homology to osteonectin and its expression is associated with systemic inflammatory diseases including rheumatoid arthritis, lupus and ulcerative colitis. Several in vitro studies have established FSTL1 as a pro-inflammatory cytokine. For example, over-expression of FSTL1 in the fibroblast-like COS7 cell line or in human U937 monocytes induced the secretion of pro-inflammatory cytokines IL-6, TNF-α and IL-1β [107].

With regards to adipose biology, FSTL1 is highly expressed in 3 T3-L1 pre-adipocytes and is implicated in their differentiation [108, 109]. Furthermore, stimulation of 3 T3-L1 adipocytes with recombinant FSTL1 inhibited insulin signalling [108] In vivo, increased adipose tissue expression of FSTL1 is reported in the leptin-deficient ob/ob mouse, and in humans serum levels of FSTL1 positively correlate with BMI [108]. Despite being expressed and secreted by human myotubes [110] no studies to date have reported the functional effects of FSTL1 on skeletal muscle insulin signalling, using either rodent or human cells.

### SPARC

SPARC (osteonectin) was first discovered as a glycoprotein secreted from bone. However, it is now known that SPARC is also expressed and secreted from adipose tissue. SPARC adipose tissue expression is increased in dietary-induced obesity in rats [111]. In humans, SPARC is secreted from adipose tissue, implicated in adipocyte differentiation and hyperplasia [112], and its expression in adipose tissue correlates with fat mass [113]. Furthermore, serum levels of SPARC are associated with insulin resistance, dyslipidemia and inflammation in patients with gestational diabetes mellitus (GDM) [114]. Mechanistically, overexpression of SPARC in 3 T3-L1 adipocytes downregulated GLUT4 expression and inhibited insulin-stimulated glucose uptake [111]. Given these data, it seems likely that SPARC would impair skeletal muscle insulin signalling. At present, these studies have not yet been conducted and so its functional role in skeletal muscle is not established.

### CTRP3

CTRP3 is a member of a family of proteins which includes adiponectin. Similarly to adiponectin, CTRP3 has been identified as an anti-inflammatory adipokine. In humans, CTRP3 levels in the serum are lower in obese subjects compared to normal-weight individuals [115, 116], and negatively correlate with markers of insulin resistance [116]. In vitro, CTRP3 inhibits LPS-induced expression of pro-inflammatory cytokines in human macrophages [117], whilst RNAi-mediated knockdown in preadipocytes increased the expression of chemokines and reduced adiponectin expression [118]. Its functional role in skeletal muscle insulin signalling has not been characterised.

### Omentin-1

Originally identified as a lectin-binding protein [119], Omentin-1 (intelectin-1) is highly expressed in visceral adipose tissue [120]. In humans, systemic concentrations and adipose tissue expression of Omentin-1 are lower in obese individuals [120] and negatively correlate with BMI and insulin resistance [120]. Furthermore, lower serum levels of omentin-1 are observed in newly diagnosed T2D patients and its secretion from human adipose tissue is decreased by both insulin and glucose [121, 122].

In vitro, studies support a role for Omentin-1 as an anti-inflammatory adipokine, which suppresses the activity of TNF-α in vascular inflammation via inhibiting p38 and JNK pathways. A role for Omentin-1 in promoting insulin sensitivity is supported by studies in human adipocytes where recombinant Omentin-1 induced AKT phosphorylation and enhanced insulin-stimulated glucose uptake [123]. Thus far, studies to determine its functional role in skeletal muscle using either rodent models or human tissue have not been reported.

### Lipocalins

Lipocalins are a functionally diverse group of proteins with a highly conserved tertiary structure that have been implicated in inflammation and immune responses. Importantly, a number of lipocalins, most notably lipocalin-2 (LCN2) and RBP4 have been associated with adipose tissue expression and obesity. Recently, a new member of the lipocalin family was identified, termed lipocalin-14 (LNC14), which in mice was found to be predominantly expressed in WAT and was downregulated in dietary-induced obese mice [124]. Furthermore, adenovirus over-expression of LCN14 in obese mice improved insulin sensitivity [124].

## Conclusions

Obesity and its associated conditions including insulin resistance and T2D are increasing globally, resulting in substantial socioeconomic costs. Since adipose tissue secretes a number of adipokines that can have both positive and negative effects on insulin sensitivity and metabolism, targeting adipokine signalling has emerged as a potential area to identify and develop novel therapeutics. Therefore, given that muscle is the major organ for insulin-stimulated glucose uptake, understanding the function and mode-of-action of such adipokines on skeletal muscle is critical.

To address this need, several in vitro functional studies have been conducted utilising myoblasts isolated from skeletal muscle tissue, and/or differentiated myotubes. However, as illustrated in this review, such studies have predominantly been conducted using cells derived from rodent skeletal muscle, which is known to have different fibre type composition and metabolic characteristics than human skeletal muscle [125]. Unfortunately therefore, much of functional and mode-of-action data generated using these rodent in vitro models may poorly translate to human skeletal muscle physiology. This is critical, since it is known that the greatest reason for late-stage failure of candidate drugs can be traced back to failure of preclinical target validation studies to translate to the clinic [126]. Furthermore, as highlighted in Table 2, the functional roles of novel adipokines such as FSTL1, SPARC and omentin-1 in mediating insulin sensitivity in skeletal muscle have yet to be studied.

To fill this gap, future studies on the expression profile of adipokines in humans need to be complimented with in vitro functional studies that utilise myoblasts and myotubes derived from human skeletal muscle biopsies or, where relevant, derived from muscle biopsies collected from T2D or insulin-resistant patients. Such studies will greatly facilitate identifying and validating novel therapeutic targets capable of improving glucose management that translate in the clinic.

## Abbreviations

FA1: Foetal Antigen 1
FSTL1: Follistatin-like 1
GDM: Gestational diabetes mellitus
GLUT4: Glucose Transporter 4
GRP78: Glucose-regulated protein
IRS-1: Insulin receptor substrate-1
LCN2: Lipocalin-2
LNC14: Lipocalin-14
Pref-1: Preadipocyte factor 1
T2D: Type 2 Diabetes
